# Fabric based printed-distributed battery for wearable e-textiles: a review

**DOI:** 10.1080/14686996.2021.1962203

**Published:** 2021-09-17

**Authors:** Adnan E. Ali, Varun Jeoti, Goran M. Stojanović

**Affiliations:** Faculty of Technical Sciences, University of Novi Sad, Novi Sad, Serbia

**Keywords:** E-textile, wearable technology, energy supply, printed battery, electrical thread, areal capacity, 50 Energy Materials, 207 Fuel cells / Batteries / Super capacitors, 206 Energy conversion / transport / storage / recovery, 700 Others: Powering Electronic Textiles and Wearables

## Abstract

Wearable power supply devices and systems are important necessities for the emerging textile electronic applications. Current energy supply devices usually need more space than the device they power, and are often based on rigid and bulky materials, making them difficult to wear. Fabric-based batteries without any rigid electrical components are therefore ideal candidates to solve the problem of powering these devices. Printing technologies have greater potential in manufacturing lightweight and low-cost batteries with high areal capacity and generating high voltages which are crucial for electronic textile (e-textile) applications. In this review, we present various printing techniques, and battery chemistries applied for smart fabrics, and give a comparison between them in terms of their potential to power the next generation of electronic textiles. Series combinations of many of these printed and distributed battery cells, using electrically conducting threads, have demonstrated their ability to power different electronic devices with a specific voltage and current requirements. Therefore, the present review summarizes the chemistries and material components of several flexible and textile-based batteries, and provides an outlook for the future development of fabric-based printed batteries for wearable and electronic textile applications with enhanced level of DC voltage and current for long periods of time.

## GRAPHICAL ABSTRACT

**Figure:**
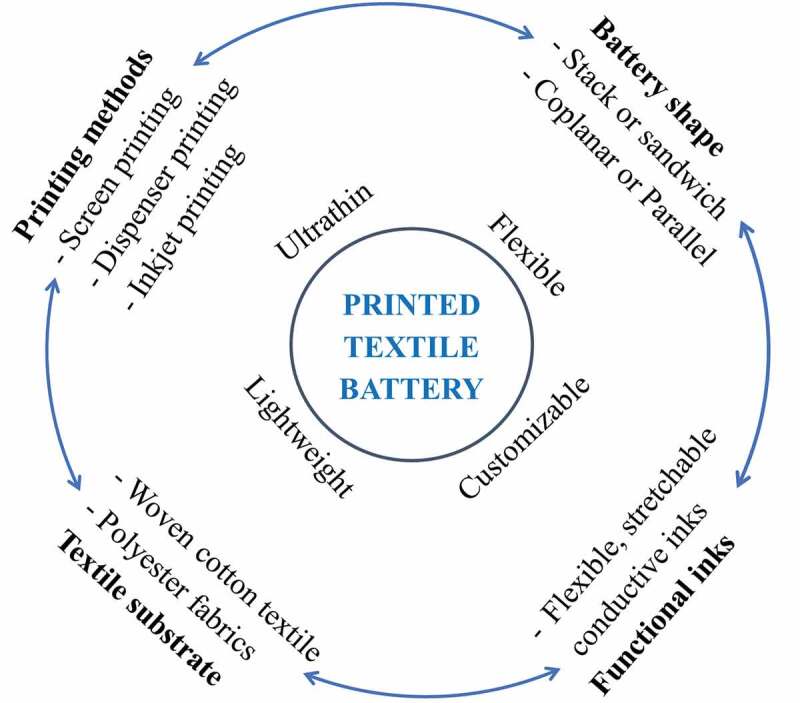

## 1. Introduction

**Figure 1.**
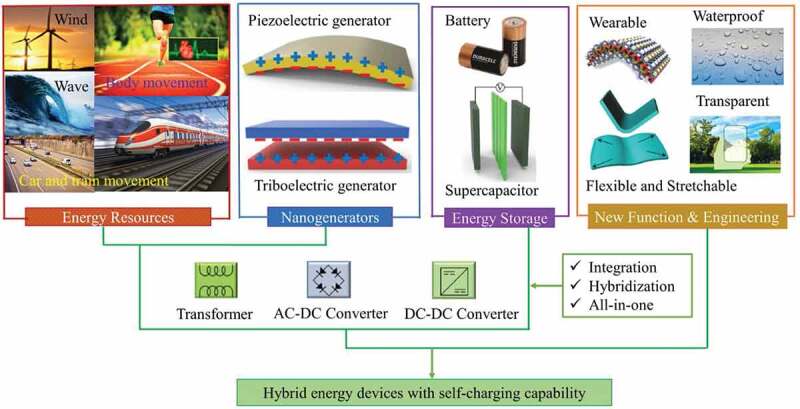
Developments in hybrid energy systems based on nanogenerators and energy storage devices through integration, hybridization, and all-in-one design for self-charging capability of future electronics. Reprinted, with permission, from [14]. © 2017, Kim et al

The past few years indicated a rapid progress towards researching the flexible electronics applied on surface or embedded with garments for various applications including healthcare, and other sensing functionalities. However, many of these devices are based on conventional electronic circuit elements and substrates like printed circuit board and, therefore, stiff and inflexible, which restricts their widespread practical applications and also ease of wearability. The future growth of these wearable electronic systems depends on effective production and use of flexible and stretchable substrate materials in order to meet an increasing demand of intelligent systems and wearables [1,2]. These new kinds of electronic textiles are knowledge based with high added value, and provide a means for incorporating electronic functions and conductive wires into fabric to make them smart wearables [3] for a wide variety of applications. However, like other electronic devices, they need a power supply; which is one of the biggest challenges that limits their commercialization [4]. The integration of power supply into textiles has its own consequences for the overall well-being of the wearer, replacement, and flexibility. Usually in conventional approaches, rigid and bulky batteries or capacitors are used as energy storage devices for electronic textiles [5,6]. After huge efforts and developments made on these traditional energy storage devices, they are now becoming smaller in size and thus more portable. To replace these rigid devices or batteries with flexible ones, studies suggest that all the battery components need to be made of flexible substrate materials. However, besides flexibility, lightweightness and comfortability, when these textile electronic devices are specifically used in a close proximity to the human body, the materials used for constructing the electronic components, such as battery, should be non-toxic and eco-friendly. This implies the conventional means of powering electronic devices have limitations and hence are not always feasible for use in e-textiles applications [7]. Power generation can also be achieved by one or combination of energy scavenging systems, such as piezoelectric or triboelectric elements [8,9] that harvest energy from human body motion; radio-frequency (RF) energy harvesting [10,11] which requires an RF source to be close to the wearer; solar energy harvesters [12,13] where energy can be collected at certain times of the day; or combination of these harvesting methods with one of the energy storage devices. Compared to other methods, the mechanical energy harvesting by using the piezoelectric or triboelectric nanogenerators is the most widely distributed harvesting method, available in all living environments including our body as shown in Figure 1. The energy collected from all the above-mentioned energy scavenging methods could be converted to a useful electrical energy; however, they show several drawbacks, usually need more space similar to traditional energy storage devices, and still require rigid electronic component that restricts the flexibility of smart fabrics that they power. Textile-based energy harvesting and storage systems could be the best alternative for powering e-textile devices.

**Figure 2.**
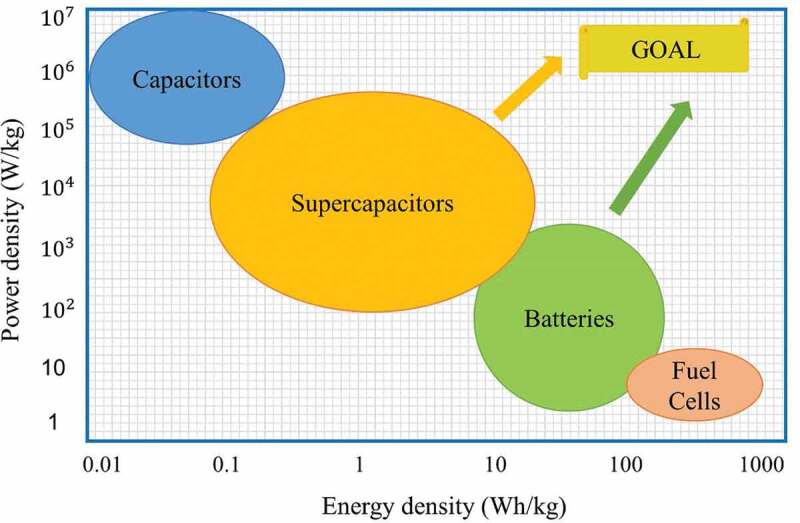
Ragone plot for the fundamental electrical energy storage devices

Studies regarding the potential application of textiles to store energy include supercapacitor [15,16] and batteries [17]. In each method of providing energy storage capability to textiles, incorporations could be performed within the textile material or built on its surface while also keeping the desirable properties of fabrics [18]. Since there is only limited free space available to connect electronic components and capacitive materials onto textiles, areal capacitance [19] is an important parameter in wearable devices. Thus, obtaining a higher mass loading [20] for every square centimeter of active material while keeping the quality of wearables is crucial for textile-based energy storage systems. Among various batteries and other energy storage devices, microbial fuel cell-based biobatteries are the least understood and developed for wearable and textile electronics applications [21]. Regardless of the energy storage system, the energy supply process occurs at the boundaries of electrode or electrolyte interface, and also the ions and electrons are transported separately. However, the energy storing and converting mechanisms are different. While batteries are suitable for high-energy applications, supercapacitors are best suited for high-power applications. Most of these devices are overqualified and expensive for applications that consume only a small amount of energy for limited time; while, microbial fuel cells are suitable for a single use, disposable electronics applications. The difference between various energy storage devices can be explained using the so-called Ragone chart as shown in Figure 2, where different energy storage methods are grouped according to their energy and power density [22]. While the energy density stands for the amount of energy stored in a given mass, the power density refers to how quickly the stored energy could be released from the devices.

In this review, different flexible and textile-based batteries, as well as printing techniques applied for smart fabrics are discussed. The chemical and material components for each battery cells are analyzed to identify a fully flexible and sustainable power supply. The present review summarizes the current-state-of-the-art flexible and textile-based battery technologies, and highlights future research directions for developing fabric-based printed batteries with enhanced level of DC power to continuously power wearable and electronic textiles for long period of time.

### 1.1. Development of electronic textiles

From the pre-industrial agricultural era to an exponentially developing information age, connected globally through networked technologies, a lot has been studied regarding the rapid changes and development of human being. Textiles arise in the first of these eras. Initially, different leaves were joined together by our predecessors to make their own styles of garment and apparel. Naturally available materials, including silk and cotton, were woven into more suitable and warm garment products. A wide range of man-made fiber materials including nylon, and Kevlar slowly came and largely improved our way of living over the last 100 years. Now with the machine age, the usual way of manufacturing the woven textile has grown to become the fabric of our lives. As a material from which is produced the interface between the outside environment and bare human body, textiles take an incomparable position in our society [23]. The evolution of information technology, in conjunction with micro-electronics and system developments, provides a unique and excellent opportunity to integrate electronic functionalities into textile materials [24]. In the last decade, the emergence of new and multidisciplinary research has been carried out in the area of textiles. The biomaterials, micro or nano-technologies as well as information technology have continued to lead the revolution. As a result, there are new possibilities and mechanisms for increased functionality of textile materials; from new structure of fibers, composites and coating at the nano- or micro-level to the actual-visible implementation of electronic functionalities in to the garments and apparel products [25]. A range of previously dissimilar technologies has now merged together, for example, the bio- and polymer chemistry meets miniaturization of computer processors in order to produce the so-called lab-on-a chip diagnostics [26]. Moreover, new types of fabric-based sensors, textile actuators, and other different electronic components are now readily available [27]. All of these make earlier dreams for a genuinely functional and smart electronics via the medium of textiles to become a reality. In addition, new conductive yarns, which can be used for weaving or knitting [28] as well as embroidering [29] into electronically functional textile materials, have also been developed, in order to provide innovative textile-based electronic products [30] acceptable to different end-users. Acceptance of electronic textiles is particularly important in the area of healthcare [31], where direct intervention is necessary, introducing new horizons for textile materials to meet actual needs and make easy interaction and monitoring of medical services through an increased comfort and convenience. The potential impact of these intelligent textiles for healthcare [32] services is significant. Risk assessment and diagnosis can be quicker and more precise, and, care and treatment will be more effective. Nowadays, intelligence is embedded in different daily objects including watches [33]. However, the fact that textile materials are highly flexible in products as well as manufacturing processes compared to other products, and can come in for direct interaction with the human body, enables them to be highly suitable for various intelligent and wearable electronics applications.

**Figure 3.**
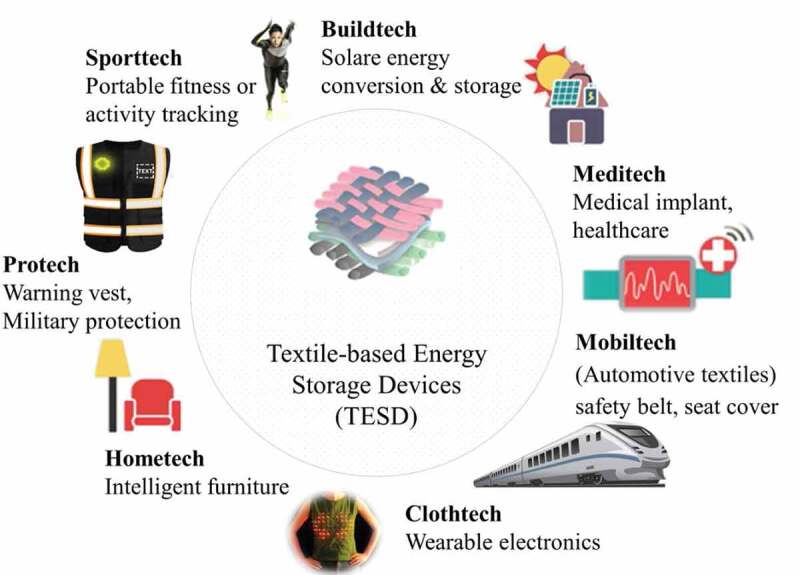
The potential applications of textile-based electrochemical energy storage devices in various areas. Reprinted, with permission, from [38]. © 2016, Wiley-VCH

The impact of electronic textiles on the economic development is expected to be extremely high according to IDTechEx report [34]. With the development of electronics and information technologies, textiles which have been used to keep us warm and comfortable, are now facing various challenges and are expected to have extra functionalities. For instance, the aim of a self-powered system, which is extremely necessary in the field of electronics, might be done when textiles are used to store different energy sources such as mechanical or solar and convert it to a useful electrical energy. These energy-storing textiles can be used to successfully and sustainably power various portable electronics including mobile phones [35]. Nowadays, electricity is everywhere and dominates our life; therefore, integrating electronic functionalities into textile materials is a promising technology to effectively use the stored energy for several applications including display and sensing systems [36]. However, there is an issue and concern about the working stability of these miniaturized electronic devices and systems. Due to their small dimension and complicated application requirements, powering them with conventional rigid batteries [37] meets difficulties in replacement or on-site charging of the batteries. Using textile for storing and harvesting energy is the best alternative to overcome this problem. This is mainly because, naturally, textiles have good mechanical stability, and large surface area, and also due to the feasibility that textile-based energy storage devices could be easily integrated into different application areas including apparel, and healthcare services as shown in Figure 3. Therefore, bringing the smart electronic textiles and wearable products to the stage of commercialization requires identifying new, low-cost and high throughput printing methods as well as compatible energy systems to successfully and sustainably power these devices.

### 1.2. Printed energy systems for e-textiles

There has been significant research interest in smart textiles and on the mechanism of powering electronic textiles which necessitates research into an energy source. Energy harvesting systems, including inductive coupling [39], thermoelectric [40], photovoltaic cells [41] and also piezo- or triboelectric materials [42], are among techniques to power wearable electronic textiles. However, all of these power harvesting methods usually need conventional battery or capacitor for storing the produced energy, and also only a small amount of energy is harvested from the surrounding environment [43]. Therefore, batteries or supercapacitors are still required in addition to energy harvesting techniques to successfully power e-textiles. Capacitors are known to store small amounts of energy but with high-power density compared to batteries. However, the rigid nature of these energy supply devices usually disturbs the typical textile haptics as well as the drape and bendability [44]. This is why textile-based batteries are of high interest in the field of wearable electronic textiles and are investigated by various research groups in [45,46]. The energy generated from the battery is due to an oxidation and a reduction reaction, which provides voltage of the battery as a result of the potential difference between these two reduction potentials.

### 1.3. Objectives of this review

This paper focuses on the critical review of different flexible and textile-based batteries, and gives a comparison between them in terms of their potential to power wearable and e-textile applications. Various printing techniques applied for smart fabrics are discussed and those applicable for thick-film deposition in order to obtain high-energy density textile battery will be identified. Regardless of the printing technology, proper adhesion of the printed layers with the substrate material is challenging to boost the scalability of printed batteries. Therefore, understanding the chemical and material components for each battery will be helpful for the future development of textile-based printed batteries with enhanced levels of DC power applicable for long periods of time.

## 2. Printed batteries for wearable e-textiles

**Figure 4.**
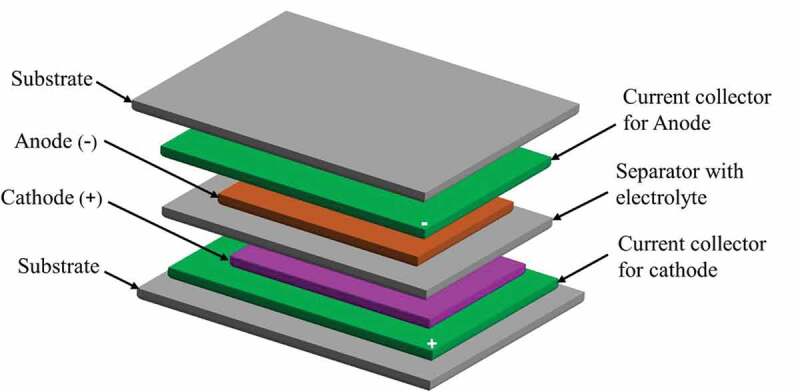
Schematic representation of a printed battery in a sandwich cell architecture, where the anode and cathode of the battery are stacked together. Reprinted, with permission, from [48]. © 2018, John Wiley & Sons

Printed batteries have received significant attention in research and industry, since they are ultra-thin, lightweight and environmentally safe while being tailorable. In order for the battery to be identified as printed-battery, the separate battery components which includes an electrolyte or separator, electrodes, and current collector, need to be manufactured using printing technologies. The design of electrodes and architecture of the battery are crucial in order to produce an efficient battery. The two most common printed battery designs are the stack or sandwich, and coplanar or parallel architectures. The stacking architecture, shown in Figure 4, consists of two electrodes, a current collector for each electrode and a separator with electrolyte, all printed on flexible substrate materials of specific thickness. The lithium-ion and zinc – manganese dioxide chemistries are the most popular batteries in printing technologies. And the stack architecture is commonly used in lithium-ion batteries, and results in a reduced internal impedance [47] due to the short distance the ions move between the two electrodes. On the other hand, the coplanar architecture, which consists of the two electrodes placed in a side-by-side arrangement unlike the sandwiched type, is most commonly applied in the design of stretchable batteries. The separator placement is optional here, and the risk of shorting during the battery stretch is very low. The general flexibility of the battery is based on the mechanical properties of separate components.

In printed batteries, mostly inks are prepared for electrode materials as well as separator or electrolyte. For each component of the battery, these inks are made up of binders, solvents and compatible filler such as lithium-based material, based on specified properties of inks. Though printing technology provides complete freedom for the manufacturing of batteries over a large area and high-volume with minimal costs [49], developing a fully functional, flexible as well as stretchable conductive ink for various battery components is critical. For battery electrodes, ink is prepared as a slurry of active materials combined to the binder and compatible solvent solutions. The rheology of ink is customized by modifying the concentrations of a binder for each printing techniques to obtain a high-energy battery. For many years, printing has been used in the textile industries to include different patterns onto fabrics for decoration purposes. In the electronic industry, printing is used for integration of various electronic functions using different micro- or nano-technology-based functional inks [50], which enables to print several devices from various substrate materials such as flame retardant (FR4) laminates and alumina for printed circuit boards [51], and polyester or polyamide for fabricating flexible circuits [52]. Printing techniques can also be extended to wearable electronic textiles and are now becoming an emerging technique for integrating electronic functionalities onto garments using electronic inks [53] that are compatible with the fabric and have the ability to combine each other without any degradation. In the case of printing on fabrics, there are many challenging requirements such as; the ink must be carefully prepared to suit fabric’s roughness and flexibility, as well as chemical compatibility and thermal characteristics of textile materials [54]. However, the printing of smart fabrics also possesses the following attractions compared to printed electronics. First and foremost, printing on fabrics provides design freedom. This implies that, anywhere on the fabrics and in any geometry, the printing layers can be deposited. Secondly, unlike the manufacturing of printed circuit boards, printing on fabrics is an additive process [55]. Therefore, it is possible to make integration of different devices with variable functionality from many different materials. The other advantage of printing on fabrics is that it benefits from an emerging and rapidly growing field of 3D printing technology [56]. Lastly, as the functional materials can only be printed and deposited where required, there is no compromise with the textile breathability and flexibility characteristics by the fabric-based printed layers [57,58].

In this section, the three most commonly used printing methods; dispenser printing, screen printing, and inkjet printing techniques are discussed. In all of these methods, developing fully functional ink for various battery components is critical, and also crucial in order to get printed-layers with appropriate and desirable properties. Moreover, the proper adhesion of layers onto the surface of substrates is also challenging for improving scalability of the printed battery. Each of these techniques have different ink properties and film thickness. Since areal capacity is related to the thickness of printed layers, thick film deposition methods are very attractive for high-energy batteries. Throughout this review, the term textile stands for those textiles or fabrics which are suitable for clothing purpose, and the two terms textile and fabrics are used synonymously.

### 2.1. Printing techniques for e-textiles

**Figure 5.**
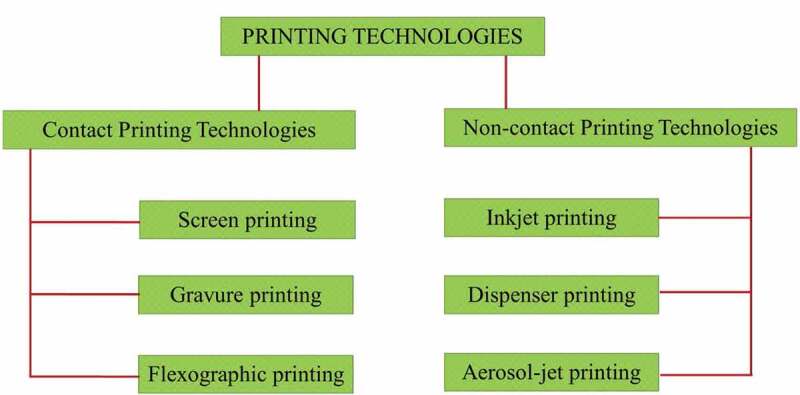
Classification of printing technologies

The incorporation of wearable technology looks set to remarkably enhance textiles in the near future. However, functionality alone is not enough for the world’s current market where the market strategies are predominated by various fashions and styles. Due to the growth of digital technologies, which influences both the design as well as fabrication of the end product, new printing technologies involved in garment construction and printing of different patterns on textile surfaces are now possible **[**59**]**. Moreover, rapid prototyping technologies, which enable the printing of different electronic components simultaneously with the construction of a product, are now developing rapidly **[**60**]**. In general, printing techniques can be classified as contact and non-contact techniques as in Figure 5. In the contact printing techniques, such as screen printing, an information on the printing plate corresponding to both image elements (i.e. that allow transfer of ink) and non-image elements (i.e. that do not allow the transfer of ink) is produced onto the substrate using inks transferred from the printing plate to the substrate by physical contact. In contrast, the non-contact printing technologies, such as inkjet printing, transfer information digitally, and do not require printing plates to transfer ink. In these printing techniques, transfer of ink is made through openings or nozzles without any physical contact to the substrate materials.

In this paper, the three common printing techniques; dispenser printing [61], screen printing [62] and inkjet printing [63] that are used to demonstrate the printing of smart fabrics [64] are described in detail. Each of these printing methods vary in terms of achievable throughput, and ink viscosity which is a very important parameter to obtain higher quality printed films. The screen-printing inks have the highest viscosity (50–5000 mPa⋅s), which is nearly 25 times higher than that of inkjet printing inks (2–25 mPa⋅s). So, for example, if the required film thickness for a given application is 20 µm, and ink viscosity is 30 Pa⋅s; then, screen-printing technology can be applied.

#### 2.1.1. Inkjet printing

In the last few years, many of the research works have focused on inkjet printing technology, which is a non-impact technology where the required image is produced on the surface of substrates using ink droplets. This technique is a promising method to fabricate polymer electronics and light-emitting diodes by depositing smart and functional materials. It consists of two operation modes: droplet on demand mode – which uses the piezoelectric actuating in order to produce ink droplets, and continuous printing mode – that uses magnetic field to produce ink droplets [65]. This printing technique has been developed but due to less successful practical applications, and highly experimental nature, it still did not show rapid technological developments compared to other print technologies. So far, only those printable materials for the realization of electronic components on regular circuit board and those low resistance conductor materials for use in printed transistors [66] have received research attention. This technology consists of two main ways of depositing ink droplets: thermal process – where the joule heating element is used to heat the ink [67], and piezoelectric process – where the inks are prepared by the deforming piezoelectric actuators which are produced by an electric field. The latter is preferable because the release as well as formation of ink droplets depend on the variation in the volume of inks due to piezoelectric actuators [68].

Inkjet printing of electronics started with printing of conductive layers on rigid materials, then later moved to printing of flexible substrate materials including papers and polyamides. Although this technology is popular in textile industries for the purpose of dyeing, obtaining higher pattern resolutions in the printing of electronic components on fabric substrates is still challenging. Some of the main concerns while conducting inkjet printing of electronic parts on textiles are due to the tight structure of textiles, diameter as well as natural porosity of fibers and yarns. Moreover, the printing inks should have to meet certain requirements, such as, lower viscosity, increased shelf-life, and lower surface tension. This technique has limited speed of printing and is commonly used to obtain thin-film layers; so, its practical application for printing thick battery electrodes is very low. However, due to its high pattern resolution, the technique is still more popular for designing and printing of small-sized batteries for various applications. Moreover, it is compatible with several rigid or flexible materials for depositing on paper or other substrates. However, this technology has several disadvantages when applied for smart fabrics [69], and usually results in a very low success compared to screen-printing techniques. As textiles have porous structures naturally, they absorb the liquid part of inks; so, there is small content of solid, compared with those inks for screen printing, to fill the rough surface of fabrics. Moreover, slight variations in the physical properties of ink will cause the print head to malfunction. Therefore, the prerequisites for inks properties, which are governed by material for inks and solvents, are very strict for inkjet printing of smart fabrics [70]. Usually, this technology allows printing a small number of fluids in the order of 1 up to 100 μL. In general, the dry thickness (d) of the ink droplets is given as (1).

(1) $$d=N_{d}\times V_{d}\times \frac{c}{p}$$

where $N_{d}$ stands for the number of ink droplets per area ($cm^{-2}$), $V_{d}$ is the volume of the inks ($cm^{3}$), crepresents the concentration of materials in the inks ($gcm^{-3}$), and p is for the density of materials in the inks ($gcm^{-3}$) [71].

#### 2.1.2. Dispenser printing

Similar to that of inkjet and screen-printing technologies, dispenser printing is also applicable in the production of intelligent fabrics and wearables. Dispenser printing technology integrates the computerized printing technique of an inkjet printer and effective performances of screen-printing ink materials. This printing method is an additive process which provides capability of printing different inks and altering the pattern designs, easily and when required, which can be specified immediately on the computer. Moreover, as the dispenser printing inks are suitable for high-volume screen-printing techniques, products printed with dispenser printers can be taken for mass production in screen printers using similar inks, unlike the inkjet printing where inks need reformulation for mass production using screen printing technology. Dispenser printing is a non-contact printing technique for smart fabrics where it is possible to print directly from the computer designs like that of inkjet printing with the same thickness of layer as in the case of screen-printing. Electronic functional inks can be prepared at nano- or microscale level by using functional particles and combining with different chemicals such as binders, solvents and surfactants. The working principle of dispenser printing is based on a pneumatic system, which allows printing of the design directly from the computer software. Dispenser printing technology has been used for the production of non-woven conducting fabrics [72], and also to print a thermoelectric nano-generator [73], capacitors and lithium batteries [74] on flexible polyamide substrate. Moreover, smart fabrics printed using this technique have demonstrated applications in various areas such as stretch and proximity sensing [75,76]. However, in all of these demonstrations, each printed layer was conducted with separate programmed printer paths, which is not suitable for many industries because it requires specific knowledge of printing and the process is complicated.

#### 2.1.3. Screen printing

**Figure 6.**
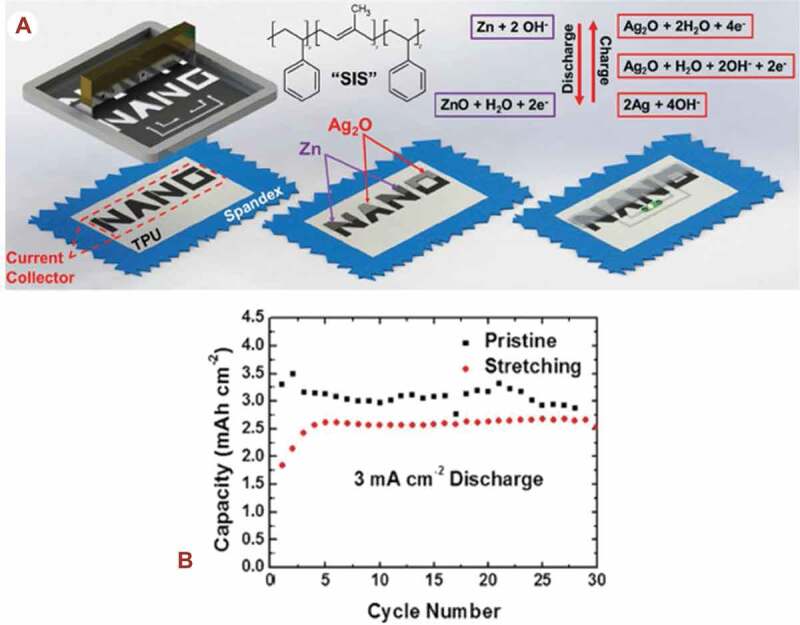
A). Stepwise screen printing of a Zn-Ag_2_O battery on a stretchable textile using a styrene-isoprene-styrene (SIS) binder. B). The discharge capacity during prolonged cycle, cycled with 3 mA h cm^−2^. (A, B) Reprinted, with permission, from [82]. © 2016, Wiley-VCH

Screen printing is an impact printing method where an ink is applied through a fine-mesh screen onto a surface, except in those areas blocked by the stencil to make them impermeable to the ink [77]. A screen is required for each functional ink, and also to define the geometry of each layer. This technique is commonly used in printed circuit boards and other applications, including printed capacitors and resonant circuits [78]. Screen printing has been used to print carbon electrodes, circuits and wearable systems for monitoring vital signs on various woven and non-woven fabrics by using on silver-based inks [79,80]. This technology is suitable for coarse textile structures such as patch antennas [81], resulting in flexible and lightweight components. And, in wearable electronics, screen printing has been used to fabricate flexible and rechargeable battery electrodes to be integrated with electronic textiles. A flexible zinc – silver oxide based rechargeable battery, shown in Figure 6(A), have been fabricated by using screen-printed conductive ink, and an extremely elastic block polymer structures the so-called styrene-isoprene-styrene (SIS) binder, which has a great potential to withstand higher loadings and maintain the required properties of battery [82]. Each battery components were printed using a low-cost, high-throughput screen printing method. This fabricated textile-based battery demonstrated a maximum reversible capacity as well as discharge current density as indicated in Figure 6(B). The mechanical properties of the printed battery was measured by using a scanning electron microscopy technique. The film thickness is determined by the mesh size and screen-printing ink properties such as low volatility and high viscosity [83]. In this printing technique, the ink from the mesh will be transferred onto the substrate, by capillary action [84], when the squeegee moves across the surface of the screen. The length of ink penetration via the capillary can be determined using the Lucas–Washburn formula [85] that describes the relationship between the length of ink penetration L(cm), & viscosity of ink δ (Pa⋅s) as shown in formula (2).

(2) $$L\propto \delta^{-1/2}$$

This relation between L and δ indicates an inverse proportionality of the length of penetration and the viscosity of ink. Therefore, viscosity of ink should be adjusted based on the type of fabric to be printed, number of ink droplets, and the printing speed in order to get maximum print quality. Unlike inkjet printing technology, the screen printing is a thick film [86] deposition technique with capabilities of roll-to-roll manufacturing; thus, applicable for large-scale production. Each printing methods have their own merits and demerits as shown in Table 1, and also different characteristics in printing, such as cost, pattern resolution and thickness. The inks for various battery components need to be designed by considering their compatibility with the printing techniques and also the electrochemical performance. The separator or electrolyte is the most difficult battery component in printing technology since the required high ionic conductivity and mechanical stability needs to be enhanced [87,88]. While high viscosity is a must for thick film and 3D extrusion printed batteries, low viscosity and surface tension must be used for thin film printed batteries [89,90].

**Table 1.** Printing technologies applied to e-textiles: resolution and advantages and disadvantages

Printing methods	Resolution, µm	Viscosity, mPa·s*	Cost	Throughput	Maintenance Requirement
Screen printing	20–100	50–5000	Low	High	Low
Inkjet printing	5–200	2–25	Low	Low	High
Flexography	30–80	1000–2000	High	High	High
Gravure printing	15	10–800	High	High	Low

mPa·s* stands for millipascal-seconds. The viscosity of ink can be measured using millipascal-seconds (mPa·s) or centipoises (cP), where 1 cP equals 1 mPa·s. The more viscous a printing ink is, the more resistant it’s to flow.

## 3. Powering wearable electronic textiles

**Figure 7.**
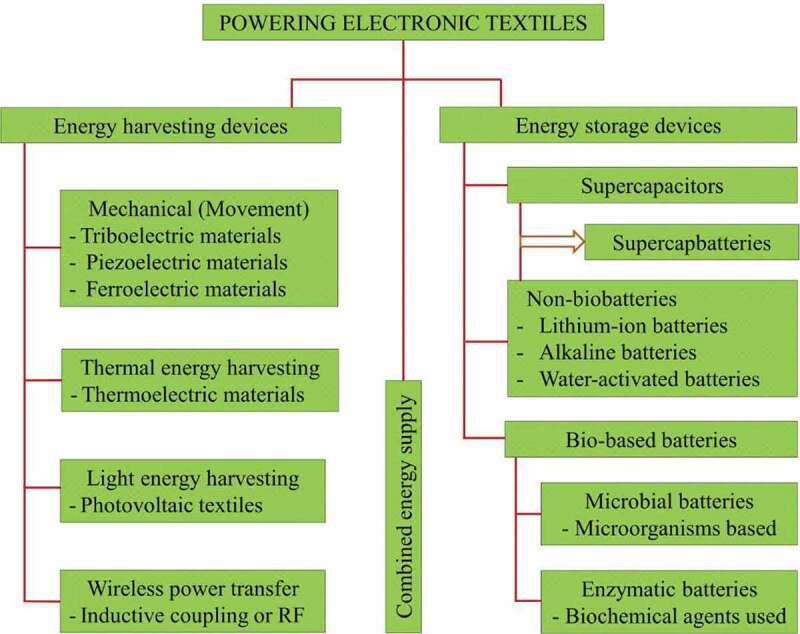
Classification of power supply mechanisms for electronic textiles and wearables

Recent progresses in wireless technology, microelectronic systems, as well as internet accesses have helped electronic objects to be interconnected, so that they communicate with another, and provide users with sufficient information to make decisions as well as to improve their life **[**91,92**]**. Towards such an environment, lightweight, wearable and flexible electronic devices in various forms such as sensors integrated with textiles to monitor health would be widely adopted. Wearable electronic textiles, besides their feel being similar to regular fabrics, have additional embedded functions including sensing, processing, storage and communication **[**93**]**, which enables them to be used in military activities, healthcare and medical textiles among others **[**94**]**. So far, only the sensing functionality of these textiles, which provides an interface between different electronic components and the end-users, is an active area of research. Therefore, smart garments and wearables might be able to monitor different physiological variables and can be useful for several applications including healthcare monitoring. Future generation of these wearable systems would be thin and consistent to human body **[**95,96**]**, so powering these devices without losing their mechanical property will be difficult **[**97,98**]** and at the same time a key, if done well, for successful commercialization of smart garments. The power supply for e-textiles can be performed through energy harvesting methods, such as photovoltaics, and also energy storing supercapacitors or batteries as given in Figure 7.

**Figure 8.**
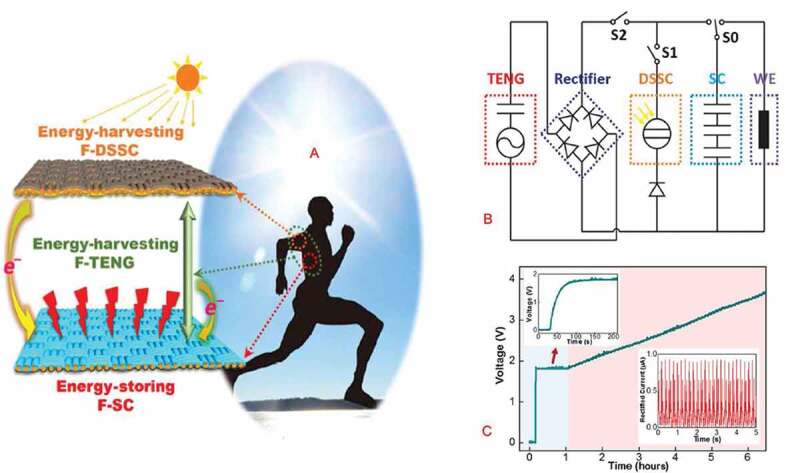
A). Self-powered textile device produced by hybridizing fiber-shaped triboelectric nanogenerators (F-TENG), dye-sensitized solar cells (F-DSSC), and supercapacitors (F-SC). B). Circuit diagram of the self-charging powered textile for wearable electronics. C). Charging curve of the F-DSSC and the F-TENG, where the light blue-shaded area corresponds to the charging curve of the F-DSSC and the light red–shaded area corresponds to the charging curve of the F–DSSC/F-TENG hybrid. The top left corner inset shows an enlarged curve during the F-DSSC charging period, and the bottom right corner inset shows the rectified short-circuit current, *I_SC_* of F-TENGs. (A, B, C) Reprinted, with permission, from [103]. © 2016, The Authors, some rights reserved; exclusive licensee AAAS. Distributed under a CC BY-NC 4.0 license

One method for powering wearable and textile electronic applications is by using the energy harvesting and wireless power transfer methods. However, many of the systems powered by the energy scavenging methods require the harvested energy to be stored until a sufficient amount of energy is available. Therefore, combining flexible energy harvesting methods with a textile-based energy storage provides the potential for sustainably and continuously power the future integrated electronic textiles and wearables. Compared to many of the harvesting techniques, the mechanical energy scavenging by using the piezoelectric or triboelectric nanogenerators is the most widely distributed harvesting method, available in all living environments including our body [99,100]. The mechanical energy harvesters generate an instantaneous power (i.e. AC current) instead of continuous power, as a result, additional rigid electrical component will be required to convert the generated power to DC power for practical application in wearable electronics and smart textiles [101,102]. For example, a self-powered system, given in Figure 8(A), has been researched, which was designed to collect solar energy from outside environment, using the fiber-shaped dye-sensitized solar cell, and mechanical energy from human body motion, using textile-based triboelectric nanogenerators, at the same time, and then store the harvested energy in a fiber-shaped supercapacitor device [103]. This all textile-based hybrid self-charging system could be easily woven into wearable e-textiles for producing smart electronic textiles. However, the mechanical energy harvesting using the triboelectric nanogenerators usually requires a rectifier circuit as shown in Figure 8(B) to convert the generated ac to direct current/power for practical application in electronic textiles. Generally, triboelectric nanogenerators have high internal impedance compared to the dye-sensitized solar cell (DSSC), and supercapacitors (SCs). Therefore, impedance matching among these three devices can be applied, as in Figure 8(C), to the system to improve the self-charging characteristics of this textile-based hybrid self-powered device.

**Figure 9.**
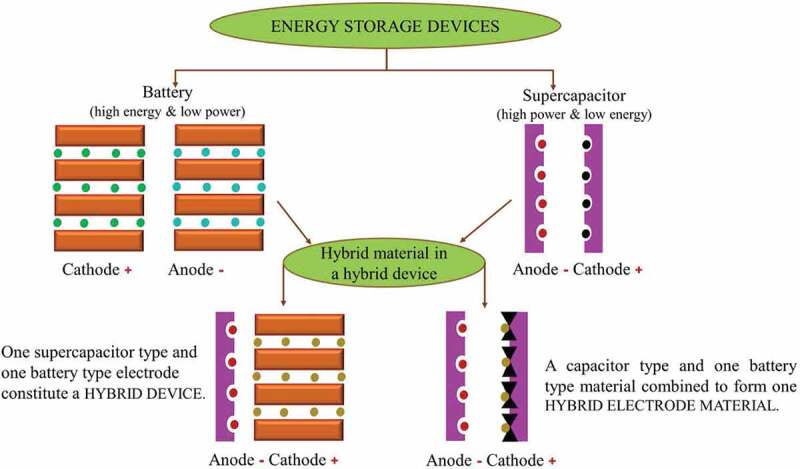
Schematic of different energy hybridization approaches between battery and supercapacitor electrodes and materials

An alternative to the implementation of energy harvesting techniques as an energy supply to wearable and textile electronics is to make use of the energy storage devices integrated with flexible and textile substrates. Energy storage devices include batteries, supercapacitors and fuel cells. Compared to both supercapacitors and fuel cells, batteries have a widely accepted market potential for applications requiring high energy storing capability. On the other hand, supercapacitors have high-power density; but, the problems of low-energy density and high self-discharging rate of supercapacitor still requires solution and hence limits the practical application of smart textile electronics. Knowing that the energy density is directly related to the working potential and capacitance of supercapacitor [104], the hybrid energy system combining the two energy storage devices, given in Figure 9, was also studied [105]. This concept of hybridizing battery and supercapacitor in a single electrode would help to improve thermodynamics and kinetics of an electrochemical reaction within a single device. And these hybrid energy devices so-called supercapbatteries are expected to have a high energy density provided by the battery electrode and also a high power density enabled by the electrode from the capacitor. However, maintaining the balance between the energy and power density requirement of the hybridized energy system is still challenging. Moreover, the design of devices with high performance does not only depend on the electrodes, but also the way electrode materials integrate with other components such as electrolyte solutions, separators and current collectors. Since the performance of the electrode materials in this hybrid device depends on both battery and capacitor components, a deep knowledge of surface chemistry in between the two energy storage devices, and also integrative material engineering needs to be explored to produce well-defined hybrid structures for enhanced transfer of charge between the electrode materials in the hybrid device.

In general, batteries have a widely accepted market potential for applications requiring high energy storing capability, than both microbial fuel cells and supercapacitors [106]. There is also a growing excitement towards the microbial fuel cell-based batteries as micro-organisms can harvest electrical energy from waste-water or biomass. The sweat produced by the human body could be used as a fuel to support bacteria viability and provide a long-term performance of these microbial bio-batteries. The biobatteries are electrical energy storage devices that can be powered by organic compounds available in glucose form used in the human body. However, these technologies are still not well-developed for wearable and textile electronic applications due to the low performance of battery, and restrictions for long-term storage of bacteria [107]. The realization of bio-batteries on textiles or paper substrate materials [108] have also been researched; but even these technologies take much time for bacteria culturing operations to generate power, and hence they are used for disposable low-power electronic applications. Integrating flexible and lightweight non-biobatteries into textile materials by fabricating them on commonly available textiles is therefore the best alternative to continuously and sustainably power wearable electronics for longer periods of time. Recently, various researches have been done in the area of textile-based batteries for powering e-textiles, but they are still far away from being considered for commercializing products. In smart wearable electronic textiles, areal capacitance is an important parameter because only a limited-free space is available for integration of electronic components and capacitive materials. Therefore, obtaining higher mass loading for every square centimeter of active materials while keeping the quality of wearables will effectively use textiles for energy storing for many applications. This is mainly because natural textiles have good mechanical flexibility, large surface area, and lightweight, and also due to the feasibility that textile-based energy storage devices could be easily integrated into different applications including garments and healthcare services. So far, the bio-based batteries and other energy supply methods have not been practically used for wearable and textile electronic applications, and hence the following sections focus on lithium batteries, liquid activated batteries, and alkaline batteries, all fabricated on textile substrates as a potential energy supply for e-textiles and wearables.

### 3.1. Textile-based lithium battery

Lithium-ion batteries (LIBs) are largely applicable in our everyday life. First commercially developed by Sony in the 1990’s, these batteries are now available everywhere in consumer electronics. These batteries are the latest in the rechargeable battery technology, and the market potential for these batteries is growing quickly with enhanced investment levels across different value chains worldwide. Currently, the rigid lithium batteries produced in metallic foil materials are powerful and have a great influence on the battery technology for various applications due to their high-energy density and longer discharge lifetime. In the last ten years, a lot has been done to develop flexible lithium-ion batteries; however, because of limited capacity and high mass per unit volume of electrode materials, enhancing the energy density while keeping the flexibility, lightweight and charge/discharge cycle stability of battery is still challenging. These batteries are attractive battery chemistries which are useful for many different electronic devices and systems because of their high-energy density and proven charging or discharging cycle time. Alhough zinc-based alkaline batteries have shown high areal capacitance [109], most of the research work has been focused on textile-based lithium batteries. This is mainly because, compared to other battery types, lithium battery provides high-energy level. Moreover, this battery is rechargeable, and can potentially be incorporated into textile materials of different forms. However, these batteries are extremely air and water sensitive, exposure to which is harmful for the safety and performance of batteries [110]. An additional disadvantage of this battery is that it contains combustible liquid electrolytes, which poses a safety risk and hence limits its practical applications for electronic smart fabrics. Although it is still a common challenge to design and develop waterproof textile-based power supply for wearable electronics and smart garments, the washability concern is more complicated in the case of lithium batteries because they consist of several materials that are extremely sensitive to air and water [111]. In general, the textile-based lithium batteries should not contain metallic current collectors as well as liquid electrolytes to be suitable for powering smart wearable and textile electronics. During charging, the lithium-ion moves from the negative electrode (anode) to the positive electrode (cathode), and the lithium metal oxides, where the metal could be manganese, iron, and so on, serve as anode and graphite is used as cathode. The charging mechanism for the complete cell is expressed as (3) and (4) for the anode and cathode, respectively.

(3) $$\left(Anode\right)LiMO_{2\;}\overset{oxidation}{⟶}Li_{1-x\;}MO_{2\;}+xLi^{+}+\;xe^{-}\;\;\;\;$$

(4) $$yC+\;xLi^{+}+\;xe^{-}\overset{reduction}{⟶}Li_{x\;}C_{y}\left(Cathode\right)$$

And, during discharging, it will be reversed; the lithium metal oxides, $LiMO_{2\;}$, become cathodes and graphite will serve as anode. Hence, the discharge mechanism for the complete cell is given as (5) and (6) for the anode and cathode, respectively.

(5) $$\left(Anode\right)\;Li_{x\;}C_{y}\overset{oxidation}{⟶}yC+\;xLi^{+}+\;xe^{-}$$

(6) $$Li_{1-x\;}MO_{2\;}+xLi^{+}+\;xe^{-}\overset{reduction}{⟶}LiMO_{2\;}\left(Cathode\right)$$

Flexible and textile lithium battery containing the LiFePO_4_ and Li_4_Ti_5_O_10_ as its cathode and anode materials, respectively, and a solid poly (ethylene oxide) (PEO) electrolyte have been researched for electronic textile applications [112]. These batteries could be recharged, and further stitched or woven as a strip onto different textile materials. And a series combination of many of these flexible lithium battery stripes, using aluminium and copper wires, were able to power up a light-emitting diode whose voltage requirement was 3 V. The PEO solid polymer electrolyte used in textile batteries needs to be flexible, and should also have high ionic conductivity. However, like that of commercially available lithium batteries, this battery still contains metallic packaging, and it is not clear how such battery packaging can be integrated in the flexible and textile-based batteries without any influence on the breathability and other desirable properties of fabrics.

On the other hand, the lithium-sulfur (Li-S) battery shows a great promise to be applicable for different flexible and textile electronic applications, due to their low mass per unit volume of electrode materials and high capacity compared to the lithium-ion batteries (LIBs). However, many of the reported batteries on the lithium–sulfur chemistry depends on using the heavy lithium foil anodes, which leads to various downsides of this battery such as low current efficiency of lithium anode and the fast dry of electrolyte solutions; low-energy density because of the increased weight of lithium foil materials and high electrolyte amounts; easy development of lithium dendrites; as well as very low flexibility [113]. To overcome this problem, many researchers focused on developing stable lithium anode and sulfur cathode with enhanced current efficiency. As a result, various plans have been described in order to obtain stable lithium metallic anode materials, including designing of solid electrolyte interfaces, and also effective sulfur cathode via different chemical improvement methods in order to lower the polysulfide’s shuttle effects, which results in a very fast fading of capacity and poor current efficiency of the battery cell. Although lithium-sulfur technology has shown rapid progress, the state-of-the-art technology still cannot avoid using high amounts of lithium anode material, which is environmentally unfriendly and more expensive. In addition, obtaining high-energy density and stable charge/discharge cycling in the flexible lithium-sulfur battery by using small amounts of lithium is still a critical problem, and hence this battery is still far from the feasible research target in order to substitute the lithium-ion technology. Therefore, an increased focus needs to be placed on different battery chemistries and associated materials to identify compatible and sustainable energy supply for electronic textiles.

### 3.2. Liquid – activated battery

**Figure 10.**
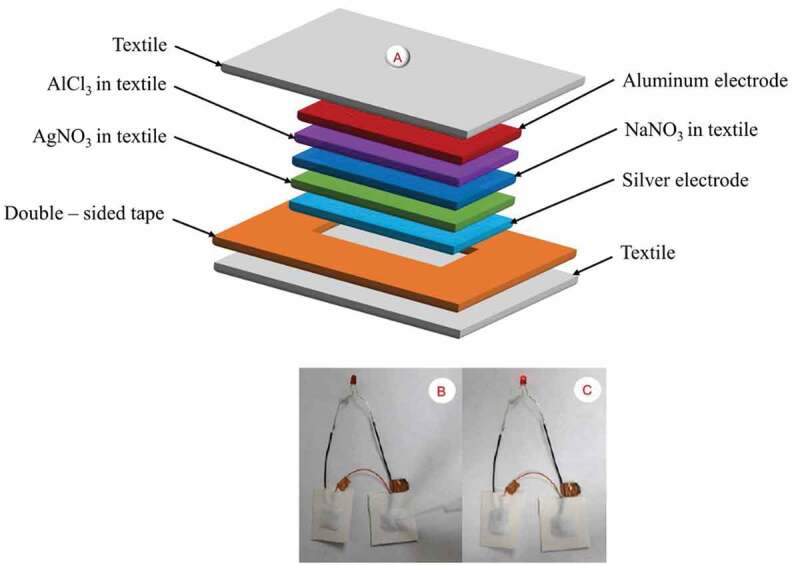
A). Schematic representation of textile-based battery including each functional layers and textile packaging. B). Two screen-printed textile-based liquid-activated batteries before activation. **C**). after activation by using 20 μL of deionized (D.I.) water and connected in series to power a 1.6 V light-emitting diode. (A, B, C) Reprinted, with permission, from [118]. © 2015, IOP Publishing. Distributed under a CC BY 3.0 license

Most commercial lithium-ion batteries are made up of rigid metallic components, which restrict their ability to be integrated with textiles and other flexible materials. Also, flexible textile-based batteries using solid polymer electrolytic have been demonstrated promising results **[**114**]**. However, all these batteries still require additional circuitry to begin and end the operation and hence they have limitations that are applicable to wearable biosensor systems. Alternately, liquid-activated batteries, which will be activated by adding liquid sample such as water to the battery cells, are designed to meet these requirements. These batteries were first developed to be used for applications that needs lower temperature, increased capacity and discharging current. This technology offers advantage for different sensor applications where liquids are used for sampling and also to activate the devices. Moreover, this technique is applicable for generating power whenever needed, so it simplifies the design as well as general operation of the devices. Water-activated batteries are classified as reserve batteries where the water is the missing element that releases the stored energy within the electrode materials. These batteries are applicable in various areas such as life jacket, emergency devices, and undersea experimentation tools. One of the traditionally known water-activated batteries are the magnesium-copper chloride, Mg-CuCl, batteries with soluble copper ions in water, which further may bring environmental risks and also damage of magnesium anode material. Similarly, the liquid activated magnesium-manganese dioxide, Mg-MnO_2_, batteries have been researched **[**115**]** and showed potential application like the Mg-CuCl batteries. It was reported that the manganese dioxide cathode materials are environmentally safe, inexpensive, unsolvable, and lightweight and hence best suited for use in the water-activated batteries **[**116**]**. However, compared to the magnesium-copper chloride batteries and magnesium-manganese dioxide, batteries gave stable but low output voltage. Also, few researchers have demonstrated fabrication of these batteries on paper and plastic substrate **[**115,116**]**, where the batteries generated an open-circuit voltage of 1.3 V and 1.2 V, respectively. And to obtain enhanced durability and easy integration with wearable textile materials, liquid activated batteries on a textile substrate were also produced by using metallic electrode materials embedded with textiles. However, it is also reported that the use of metallic film on the design of the battery cell restricts the robustness as well as flexibility of the devices **[**117**]**. Alternatively, a textile-based water activated battery was studied **[**118**]**, where the battery demonstrated potential application of powering a light-emitting diode. This fabric-based battery, which needs water to be added for the activation process, was produced by using screen-printing of the silver, Ag, and aluminum, Al, electrode pairs. However, the fabrication process consists of many textile layers stacked up together to form the battery, which increases the weight of the device and is not comfortable to integrate with wearables. The battery contains three layers of a commercially available cotton fabric **[**119**]** inserted between two metallic electrode materials, which functions as anode and cathode as in Figure 10(A). The dry electrolytes for the battery cells contain two layers of woven fabric sequentially soaked into silver nitrate, AgNO_3_, and aluminum chloride, AlCl_3_, compounds, and the other one is soaked into sodium nitrate, NaNO_3_, metal to be used as a salt bridge **[**118,120**]**. Thus, when water is added to a battery, the dry electrolytes will be hydrated and hence an electrochemical reaction will be produced. The general chemical reactions for the battery cells at the two electrodes are expressed as in (7) and (8), respectively.

(7) $$Ag\to Ag^{+}+e^{-}$$

(8) $$Al^{3+}+3e^{-}\to Al$$

A single battery cell produces a maximum potential of 1.3 V. Two of these printed battery cells combined in series before activation in Figure 10(B), and after activation by using 20 μL of deionized (DI.) water shows their potential to successfully powerup a 1.6 V light-emitting diode for a maximum of 30 minutes as shown in Figure 10(C). A big plus of this battery is its potential for generating power when required and also its compatibility with different liquid types including biofluids and buffer solutions besides water, widening its biosensing application which requires numerous individualistic measurement approaches for longer periods of time. Moreover, the method demonstrates the potential application of liquid-activated textile batteries that could be integrated into wearable fabrics for various biosensing applications. However, the battery as manufactured is in its dry form, and water needs to be added to start or activate the battery. If no water is added to the battery, it will remain inactive for unlimited periods of time. Moreover, this battery contains a number of fabric layers that may add an extra weight, and hence increase the overall thickness of the printed battery.

### 3.3. Textile – based alkaline batteries

Alkaline chemistries are the predominant primary batteries which drives its energy from the zinc metal and manganese dioxide chemical reactions. In the basic environment, these batteries have a pH of approximately 14. The basic electrolytes for these battery chemistries are potassium hydroxide, KOH or sodium hydroxide, and NaOH, and hence it is named as alkaline battery. The amount of hydroxide ion, OH^−^ consumed or produced, during discharge, is equal. These batteries have characteristics of long discharge cycle life, long storage time, and leakage proof. The most commonly used alkaline batteries applied for smart textiles and wearables consists of the zinc-manganese dioxide, Zn-MnO_2_, and the monovalent silver oxide-zinc, Ag_2_O-Zn, batteries.

#### 3.3.1. Manganese oxide – zinc based battery

**Figure 11.**
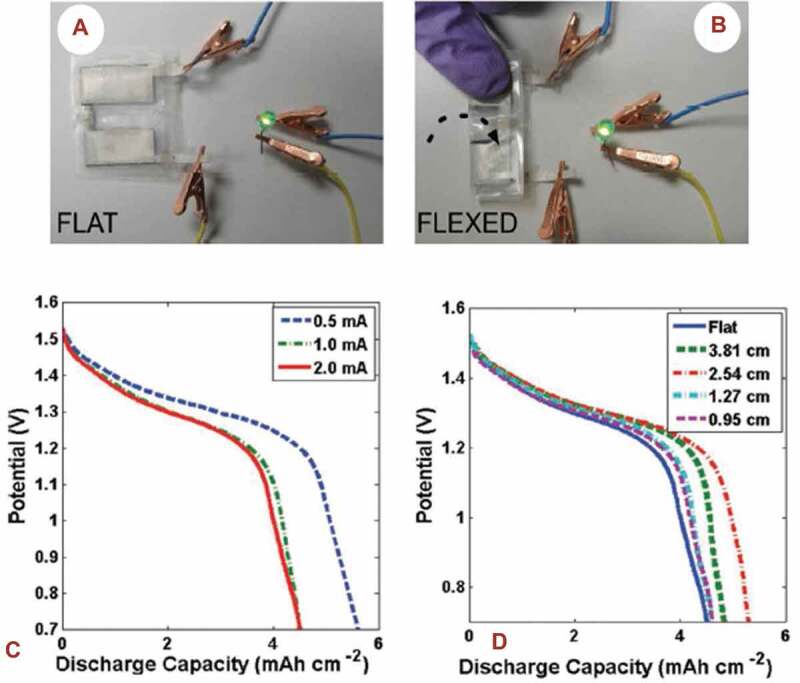
Characterization of the flexible Zn-MnO_2_ based alkaline printed battery. A). Demonstration of two flexible batteries connected in series to power a green light-emitting diode. B). The flexible batteries connected in series were able to power a green light-emitting diode when flexed to a bend radius of 0.3 cm. C). Discharge profile of the flexible battery when discharged at 0.5, 1, and 2 mA when flat. D). Discharge profile of the flexible battery when flexed to different radii of curvature while discharging at 1 mA. (A, B, C, D) Reprinted, with permission, from [122]. © 2011, Wiley-VCH

The manganese dioxide – zinc battery is an alkaline battery, that contains the zinc anode, manganese dioxide cathode, and an electrolyte solution composed of potassium hydroxide, KOH. This battery chemistry has a higher gravimetric as well as volumetric energy density than the zinc-carbon chemistry, but it is more expensive. The MnO_2_ -Zn battery is not rechargeable; this is due to the fact that during plating the zinc ‘dendrites’ will be formed and also because of the corrosive nature of the electrolyte; this battery has not been widely applied for textile-based flexible electronic applications. This alkaline battery is environmentally safe and hence applicable for most of the flexible printed batteries. The printed, nontoxic, flexible and high-energy manganese dioxide-zinc-based battery was fabricated [121], where the battery electrode materials were produced by using a solution-based integrating method with a polymeric gel electrolyte material and silver inks as a current collector. These batteries produce an output voltage of about 1.52 V, and two of these printed batteries combined in series demonstrated their potential to successfully power a light-emitting diode as shown in Figure 11(A,B), whose voltage and current requirements were in the range of 1.9 to 2.4 V and 4–32 mA, respectively. The discharge characteristics of batteries, given in Figure 11(C,D), were analyzed in different bending situations. In general, the half-cell reaction due to the zinc-manganese dioxide chemistry, shown in (9) and (10) produces ZnO and MnOOH at the anode and cathode, respectively.

(9) $$Zn+2OH^{-}\to ZnO+\;H_{2}O+2e^{-}$$

(10) $$MnO_{2\;}+H_{2}O+\;2e^{-}\;\to MnOOH+OH^{-}$$

In this battery chemistry, the output voltage decreases with the depth of batter’s discharge cycle life. One of the drawbacks for this battery because a constant voltage is desired for the circuit. Thus, the sloping open-circuit voltage with an increasing discharge time needs to be considered for manganese oxide-zinc based circuit applications; hence, the MnO_2_-Zn based printed alkaline battery chemistry has not been widely expanded.

#### 3.3.2. Silver oxide – zinc based battery

Among several commercially available flexible batteries, aqueous zinc chemistries were effective for the development of low-cost, high throughput products [123,124]. Due to its high theoretical capacity, low cost compared to lithium-ion batteries, and safe battery chemistry, the zinc anode battery has been researched for the light-weight and flexible battery market [125,126]. However, usually these batteries are not rechargeable and also have high impedance, so their practical application is limited to low-power, disposable electronics. To address this problem, different studies have focused towards developing a printed, rechargeable zinc battery with enhanced performance [127,128]. The monovalent silver oxide-zinc, Ag_2_O-Zn, chemistry is one of the alkaline batteries which have received significant research attention because of their rechargeable chemistry and tolerance to high discharge current [129,130]. These batteries have a high power as well as a high energy density, which is due to the fact that zinc is the most electropositive element suitable for aqueous solutions [131]. The success and widespread acceptance of electronic textiles depends on their ability to be manufactured through existing textile technologies and use the textile area for storing as well as harvesting energy. Integrating an electrochemical energy storage functionality onto textile fabrics using the monovalent silver oxide-zinc based chemistry was researched [132]. The fabricated textile-based battery generates DC voltage and current when moistened by ionically conducting liquids, such as sweat, readily available on our body which serves as an electrolyte. The electrochemical cell consists of the monovalent sliver oxide, Ag_2_O, cathode and the zinc anode electrodes deposited onto polyester fabrics using a screen-printing technology. The chemistry of these batteries involves an oxidation-reduction reaction of the two electrode materials, where the monovalent silver oxide cathode and the zinc anode electrodes are reduced and oxidized as shown in (11) and (12), respectively.

(11) $$Ag_{2}O+H_{2}O+\;2e^{-}\;\to 2Ag+\;2OH^{-}$$

(12) $$Zn+2OH^{-}\to ZnO+\;H_{2}O+\;2e^{-}\;$$

**Figure 12.**
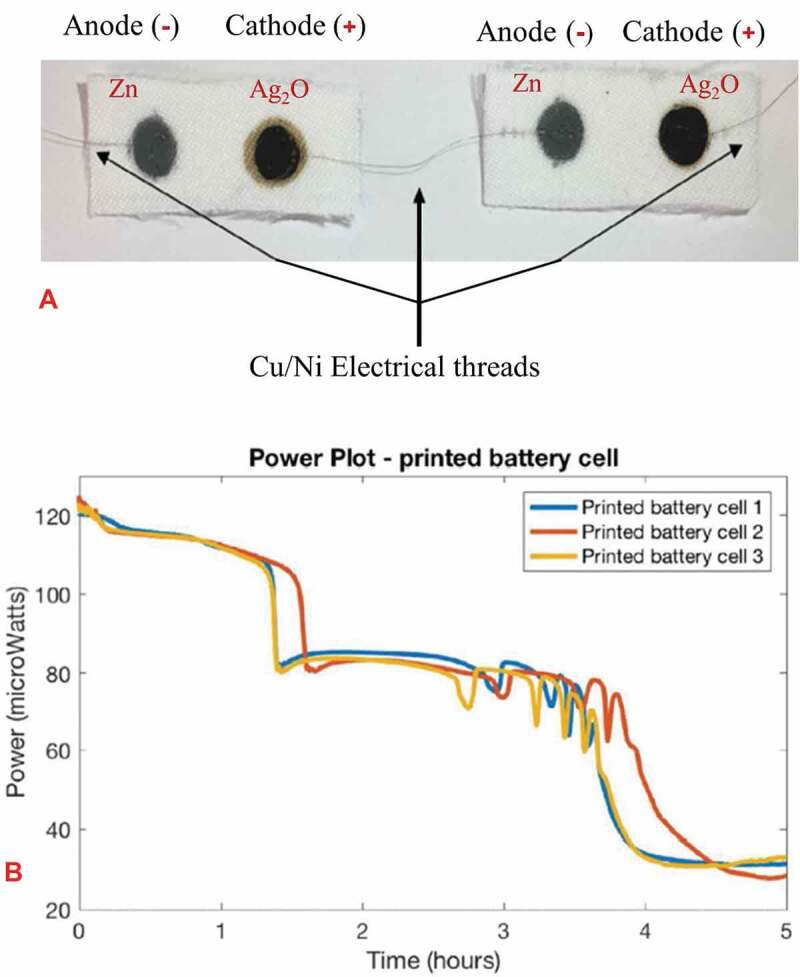
A). Two screen printed monovalent silver oxide -zinc (Ag_2_O-Zn) alkaline battery cells on two separate fabrics and combined in series using flexible Cu/Ni electrical threads for maximum electrical contact. B) Power discharge curve showing the power generated as a function of time for different battery cells connected in series at a discharge current of 100 µA. (A, B) Reprinted, with permission, from [132]. © 2018, IEEE

The current collectors for this printed battery cell include fully flexible and electrically conducting copper (Cu), and nickel (Ni) threads [133,134], which are also used to combine many of these printed batteries in parallel or series and achieve the desired level of DC power according to the sensorial or application requirements. A highly non-reactive thermoplastic fluoropolymer, also called polyvinylidene fluoride (PVDF), binder in a solvent called n-methyl-2-pyrrolidone (NMP) was added to the Zn or Ag_2_O powder to prepare a conducting paste or ink that can be screen printed onto the fabrics. And a single printed battery cell in a 10 M NaOH conventional electrolyte generates a potential of 1.46 V, and approximately 80 µW of power. As a proof-of-concept, two of these screen-printed batteries onto textile fabrics combined in series as shown in Figure 12(A), using flexible Cu/Ni electrical connections, could powerup a digital thermometer whose voltage and current requirements were 1.5 V and 12.5 µA, respectively. The power generation capability, and discharge cycle life of the alkaline battery, Figure 12(B), in a 10 M NaOH electrolyte, were analyzed using a standard electrochemistry technique called galvanostatic cycling.

## 4. Discussion

In general, the monovalent silver oxide-zinc, Ag_2_O-Zn, based printed battery demonstrated many advantages compared to lithium batteries as well as the MnO_2_-Zn based alkaline battery, to develop printed textile batteries. The energy density of the monovalent silver oxide-zinc battery is a bit smaller but still close to that of the lithium batteries. This is mainly due to the high output voltage as well as the large columbic capacity of the battery. However, this energy density could be enhanced by using the divalent silver-oxide (AgO) cathode, which provides greater operating voltage and higher energy density than the monovalent silver oxide-zinc battery. Currently, flexible batteries are constructed from active materials of very low thickness, due to which they have low capacity compared to traditional batteries. Due to its high theoretical capacity and low-cost, aqueous zinc-based batteries have been researched for the light-weight and flexible battery market. However, usually these batteries are not rechargeable and have high impedance; hence, they are limited for low-power, disposable electronics applications. The performance of batteries can be characterised by various parameters such as cycle life, and voltage/capacity of the battery (Table 2). The term cycle life refers to the number of complete charging/discharging cycles that a battery could perform before it starts to decrease its performance, usually before its nominal capacity falls below 80% of the initial rated value. And the energy content of batteries is defined by the specific energy per watt-hour per kilogram (Wh/kg), and energy density, in watt-hour per liter (Wh/L). Similarly, the rate of capability of the batteries can be defined by the specific power expressed in watts per kilogram (W/kg), and power density in watt per liter (W/L).

**Table 2.** Summary of merits and demerits for different batteries in terms of safety, capacity, and cycle life

Types of batteries	Specifications/comparisons				Refs.
	Safety	Maintenancerequirement	Cycle life	Capacity/ Voltage	
Textile	Non-hazardous	Routine	Low-self	10^−2^ to 10^−1^ mAh/cm,	
lithium	chemicals	maintenance	discharge and	3.2 to 11.2 mWh/g.	[135],
battery		required	long shelf-life	A single cell produces 0.32 to 0.5 V under a lithium iodide film.	[136]
Water-	Completely	No	Negligible self-	A single cell produces	
activated	safe relative	maintenance	discharge rates	1.3 V using various liquids	
battery	to others	requirement		such as de-ionized water, tap water, phosphate buffered saline, and artificial sweat.	[118]
Alkaline batteries (Ag2O-Zn, MnO2-Zn)	Safe compared to lithium batteries	Fewer maintenance are required to ensure good performance	Low self-discharge compared to zinc-air battery	A single cell Ag_2_O-Zn battery generates 1.46 V, in a 10 M NaOH, and ~80 µW for 3 hours at 100 µA discharge current	[132]
				A single cell MnO_2_-Zn battery produces 1.52 V, and a discharge capacity of 5.6 mAh cm^−2^ when discharged at 0.5 mA.	[121]

Table 2 summarizes the properties of textile batteries such as open voltage and capacity, as well as the advantages and disadvantages of each type of batteries with regard to the charge/discharge cycle life, safety, and maintenance requirements. Among other types of batteries, the lithium-ion battery has received research attention due to its high-energy density. Usually, the lithium batteries contain flammable electrolyte solutions which poses safety concerns. However, the presented textile-based lithium battery is based on non-hazardous chemicals and provides a higher linear capacity, which depends on the volume of electrolyte solutions present in the textile fiber channel. This implies that the larger the volume of electrolyte solutions in the fibers, the more the capacities of the battery will be. Also, this textile battery also has specific energy ranging from 3.2 mWh/g to 11.2 mWh/g, depending on the discharge current and internal volume of the textile fibers channel. As a proof-of-concept, several of these textile lithium batteries were used to power up different electronic devices including a light-emitting diode where the current and voltage requirements were 20 mA and 3 V, respectively. These batteries contain aluminum and copper wires inserted into the textile structures by drawing process, which limits its performance; but still they could be easily integrated with different woven textile materials for various applications. Further improving the performance of these batteries, by optimizing the connection between textile fibers could find their potential application in the e- textiles industry.

Alternately, a liquid-activated battery, which turns ‘on’ upon addition of a liquid sample to the battery cell are presented to meet some of the requirements for e-textiles and wearable applications. These batteries offer advantages for some sensory applications where liquids such as water are used for sampling and activating the devices. Using different testing liquids such as deionized (DI.) water or phosphate buffered saline (PBS), a single cell produces a potential of 1.3 V. As a proof, dual-cell printed batteries were combined in series, and powerup a 1.6 V light-emitting diode for about 30 minutes. In these types of batteries, instantaneous activation is required, and hence they offer a unique opportunity of generating power supply on-demand, so it simplifies the design as well as general operation of the devices. Repeated re-activation of the battery may result in a quicker voltage drops; hence this technique suffers from a non-uniformity and poor stability concerns. Moreover, the poor contact between the textiles and electrode materials contributes to a higher internal impedance; the higher the resistance, the lower the performance of the battery against high discharge currents. When the water is evaporated, the electrochemical reactions will come to an end, and the battery will be turned ‘off’ automatically, so they are considered as reserve batteries. Considering that the sensing measurements usually require a short period of time, these batteries can provide enough energy for many independent measurements over long durations. These batteries could be easily integrated into different textiles with optimum flexibility and indefinite shelf-life before activation, which is highly important for emergency applications.

The monovalent silver oxide-zinc, Ag_2_O-Zn, based alkaline batteries printed on fabrics are presented here, which are safe and can be easily activated by ionically conducting liquids available on our body such as sweat. These screen-printed batteries are rechargeable unlike the manganese dioxide-zinc chemistry, MnO_2_-Zn, insensitive to air and water unlike the lithium-ion batteries, have good tolerance to high discharge current, and they behave like regular fabrics, which is crucial for electronic textiles. This is because the success and widespread acceptance of electronic textiles depends on their ability to be manufactured through existing textile technologies and use the textile area for storing as well as harvesting energy. These batteries were fabricated using a low-cost and high throughput screen-printing technology for enhanced flexibility in wearable and e-textile applications. A single printed battery produces a potential of 1.46 V, and further incorporation of engineering concepts into the battery cell enables voltage or current boost depending on the application requirements. As a proof, two of these screen-printed fabric-based batteries combined in series, using flexible electrical connections, could powerup a digital thermometer whose voltage and current requirements were 1.5 V and 12.5 µA, respectively. However, these monovalent silver oxide-zinc, Ag_2_O-Zn, batteries have high impedance, which results in low battery capacity and limited cycle life. Therefore, an increased focus needs to be placed on different battery chemistries and associated materials to overcome this problem and scale DC voltage and current for various e-textile applications.

## 5. Conclusions and future work

In the past few years, there has been significant research interest in the area of wearable and textile electronics as they remove the need for extra carriage of various devices. These wearable electronic devices have some important requirements such as flexibility, lightweightness and comfortability, among others. However, though there is progress in developing wearable and textile electronic devices, powering them still remains a challenging task. Usually, the current energy supply devices are constructed from rigid and bulky materials and often require more space than the devices they power. Therefore, energy supply for the next generation of flexible and smart textile electronic devices is key to the overall functionality of electronic textiles and wearables. Due to their high gravimetric and volumetric density as well as suitable power density for most applications, batteries are found to be the best alternatives to power electronic textiles. Printing technologies have a greater potential to fabricate light-weight and low-cost energy supply devices, by depositing different battery components with printable inks, that can be easily integrated with various textile electronic devices. Moreover, the printing processes are economical, can be customised easily, and also play a key role for high-volume roll-to-roll production of flexible batteries. Batteries can be printed on the fabrics and can also be adjusted according to the device’s mechanical as well as power requirements. Different printed flexible power supply devices have been reported in the literature. However, the current flexible batteries are also constructed from materials of very low thickness, which again reduces the areal capacity of the batteries. Therefore, there is a need to develop flexible, lightweight textile-based batteries without any rigid electrical components to continuously and sustainably power electronic textile devices. In this review, different printing techniques, including screen printing, applied for electronic textiles and functional fabrics are discussed in detail. In all the printing techniques, the proper formulation of ink is a key to obtain sufficient electrical as well as mechanical properties of printed layer materials. Moreover, to enhance the scalability of printed batteries, proper adhesion of printed layer materials to the substrate is also very critical. Various battery chemistries and material components fabricated on textile substrates, such as lithium batteries, water-activated batteries and alkaline batteries, and their merits and demerits in terms of capacity or voltage, maintenance requirements, cycle life and safety are described. Each type of battery demonstrates their potential application to power different electronic devices of specific voltage and current requirements. Further incorporation of engineering concepts, using flexible electrical connections, to these batteries would help to boost the level of voltage or current depending on the application requirements. Therefore, batteries with the capability of generating high voltages are very important in e-textile applications, because the textile electronic circuits are generally designed to have a low-power consumption and a high input impedance; however, a lower impedance is desirable for component interconnections.

In general, the success of printed flexible and textile batteries depends on the development of new active materials for all the battery components; the higher the energy density of active material, the more enhanced the areal capacity. Thus, achieving high-energy density and areal capacity of battery without any increment in the overall thickness of active layers is a very critical problem in the development of textile and flexible batteries. Among several commercially available flexible batteries, aqueous zinc-based chemistries have been researched for the lightweight and flexible battery market. This is mainly due to the fact that the zinc anode battery has high theoretical capacity and low-cost compared to the lithium-ion batteries, and also due to its safe battery chemistry. For the textile-based lithium batteries to be suitable for wearable and smart electronic textiles, they should not contain metallic current collectors and liquid electrolyte solutions. Although it is still a common challenge to design and develop waterproof power supply for wearable and textile electronic devices, the washability concern is more complicated in the case of lithium batteries because they contain several chemicals that are extremely sensitive to air and water. Water-activated batteries offer the advantage of generating power on-demand, so it simplifies the design process and reduces its size. However, these batteries suffer from poor stability due to repeated re-activation process. On the other hand, the monovalent silver oxide-zinc, Ag_2_O-Zn, based textile battery, fabricated by depositing the zinc anode and monovalent silver oxide cathode onto textiles, behave like regular fabrics and can be directly integrated with different textile electronic devices. The battery, as manufactured, is in its dry form and later activated by bodily available liquids such as sweat. However, the battery showed limited cycle life and low capacity, which might be due to the poor electrical conductivity and high input impedance. Another species of silver oxide the so-called divalent silver oxide (AgO), offers comparably higher theoretical capacity, but it also has disadvantages of dual-voltage discharge curves and greater instability in alkaline solutions [137,138]. However, once this problem is fixed, the divalent silver oxide (AgO) cathode can provide much higher theoretical capacity and lower impedance, i.e. better battery performance against high discharge current. Therefore, optimization of related materials, impedance characteristics, and interconnections between the battery cells will be helpful for the future development of textile-based printed batteries with enhanced level of DC voltage and current applicable for longer period of time.
